# miR-22 and miR-205 Drive Tumor Aggressiveness of Mucoepidermoid Carcinomas of Salivary Glands

**DOI:** 10.3389/fonc.2021.786150

**Published:** 2022-02-09

**Authors:** Erika Naakka, Mateus Camargo Barros-Filho, Shady Adnan-Awad, Ahmed Al-Samadi, Fábio Albuquerque Marchi, Hellen Kuasne, Katja Korelin, Ilida Suleymanova, Amy Louise Brown, Cristovam Scapulatempo-Neto, Silvia Vanessa Lourenço, Rogério Moraes Castilho, Luiz Paulo Kowalski, Antti Mäkitie, Vera Cavalcanti Araújo, Ilmo Leivo, Silvia Regina Rogatto, Tuula Salo, Fabricio Passador-Santos

**Affiliations:** ^1^Department of Oral and Maxillofacial Diseases, University of Helsinki, Helsinki, Finland; ^2^Translational Immunology Research Program (TRIMM), University of Helsinki, Helsinki, Finland; ^3^Centro Internacional de Pesquisa (CIPE) – A.C.Camargo Cancer Center, São Paulo, Brazil; ^4^Hematology Research Unit, Department of Clinical Chemistry and Hematology, University of Helsinki, Helsinki University Hospital Comprehensive Cancer Center, Helsinki, Finland; ^5^Department of Oral Pathology, Faculdade São Leopoldo Mandic, Campinas, Brazil; ^6^Molecular Oncology Research Center, Barretos, and Diagnósticos da América (DASA), Barueri, Brazil; ^7^Department of Pathology, A.C.Camargo Cancer Center, São Paulo, Brazil; ^8^Department of General Pathology, Dental School, University of São Paulo, São Paulo, Brazil; ^9^Department of Periodontics and Oral Medicine, University of Michigan School of Dentistry, Ann Arbor, MI, United States; ^10^Department of Head and Neck Surgery and Otorhinolaryngology, A.C.Camargo Cancer Center, São Paulo, Brazil; ^11^Department of Head and Neck Surgery, University of Sao Paulo Medical School, São Paulo, Brazil; ^12^Department of Otorhinolaryngology – Head and Neck Surgery, University of Helsinki and Helsinki University Hospital, Helsinki, Finland; ^13^Research Program in Systems Oncology, Faculty of Medicine, University of Helsinki, Helsinki, Finland; ^14^Division of Ear, Nose and Throat Diseases, Department of Clinical Sciences, Intervention and Technology, Karolinska Institute and Karolinska Hospital, Stockholm, Sweden; ^15^Institute of Biomedicine, Pathology, University of Turku and Turku University Hospital, Turku, Finland; ^16^Department of Clinical Genetics, University Hospital of Southern Denmark, Vejle, Denmark; ^17^Institute of Regional Health Research, University of Southern Denmark, Odense, Denmark; ^18^Department of Pathology, Helsinki University Hospital, Helsinki, Finland; ^19^Cancer and Translational Medicine Research Unit, University of Oulu, Oulu, Finland; ^20^Medical Research Center, Oulu University Hospital, Oulu, Finland

**Keywords:** mucoepidermoid carcinoma, salivary gland tumor, head and neck cancer, oral cancer, transcriptomic analysis, miR22, miR205, microRNA

## Abstract

**Objectives:**

To integrate mRNA and miRNA expression profiles of mucoepidermoid carcinomas (MECs) and normal salivary gland (NSGs) tissue samples and identify potential drivers.

**Material and Methods:**

Gene and miRNA expression arrays were performed in 35 MECs and six NSGs.

**Results:**

We found 46 differentially expressed (DE) miRNAs and 3,162 DE mRNAs. Supervised hierarchical clustering analysis of the DE transcripts revealed two clusters in both miRNA and mRNA profiles, which distinguished MEC from NSG samples. The integrative miRNA-mRNA analysis revealed a network comprising 696 negatively correlated interactions (44 miRNAs and 444 mRNAs) involving cell signaling, cell cycle, and cancer-related pathways. Increased expression levels of miR-205-5p and miR-224-5p and decreased expression levels of miR-139-3p, miR-145-3p, miR-148a-3p, miR-186-5p, miR-338-3p, miR-363-3p, and miR-4324 were significantly related to worse overall survival in MEC patients. Two overexpressed miRNAs in MEC (miR-22 and miR-205) were selected for inhibition by the CRISPR-Cas9 method. Cell viability, migration, and invasion assays were performed using an intermediate grade MEC cell line. Knockout of miR-205 reduced cell viability and enhanced *ZEB2* expression, while miR-22 knockout reduced cell migration and invasion and enhanced *ESR1* expression. Our results indicate a distinct transcriptomic profile of MEC compared to NSG, and the integrative analysis highlighted miRNA-mRNA interactions involving cancer-related pathways, including PTEN and PI3K/AKT.

**Conclusion:**

The *in vitro* functional studies revealed that miR-22 and miR-205 deficiencies reduced the viability, migration, and invasion of the MEC cells suggesting they are potential oncogenic drivers in MEC.

## Introduction

Mucoepidermoid carcinoma (MEC) is the most common salivary gland malignancy in major and minor glands, and the most common salivary gland cancer affecting pediatric patients (1). The clinical behavior is variable, ranging from indolent locally infiltrative lesions to highly aggressive and metastatic lesions (2, 3). The widely used histological grade system stratifies MECs into low, intermediate, or high-grade (I, II, or III, respectively) according to histologic characteristics (1, 4–6). Histologic grade and TNM status are commonly used parameters for treatment planning. Treatment of low- and intermediate-grade tumors is based on complete surgical removal of the tumor, while there is no consensus regarding the guidelines for intermediate histologic grade (2, 7–10). In high-grade MEC, the treatment is generally surgery, followed by postoperative radiotherapy. The survival rates for low-grade MEC is over 90% at 10 years, while 70% of intermediate-grade and only 25% of high-grade MEC patients are alive after 10 years (1).

The recurrent chromosome translocation t(11;19) with the resulting *CRTC1-MAML2* fusion oncogene has been described in 60-90% of MECs (10–17). The fusion transcript has been found specific for MECs when comparing with other types of salivary gland tumors (17). *CRTC1-MAML2* has also been considered a prognostic marker (18–20), although its use in prognostication has been questioned (12, 21).

The gene expression profile of MECs has been reported in two studies in which the authors investigated a few MEC cases and compared the differentially expressed (DE) mRNA transcripts with other salivary gland tumors (22, 23).

miRNA expression studies were performed on a few MEC samples focusing on specific gene/miRNA pathways, such as angiogenesis, mast cell activation, and apoptosis (24, 25). In six MEC and three normal salivary gland samples, Binmadi et al. reported 68 DE miRNAs (26) [25]. Among them, miR-302a was the most upregulated and miR-885-5p the most downregulated miRNA (26).

Here, we investigated mRNA and miRNA expression profiles of 35 fresh-frozen MECs and six normal salivary gland tissue samples, followed by an integrative miRNA-mRNA analysis to select potential drivers. In an intermediate grade MEC cell line (UM-HMC-2), we used the CRISPR/Cas9 method to knock down two miRNAs (miR-22 and miR-205) overexpressed in MEC tissues, with the aim of analyzing their role as oncogenic drivers in MEC.

## Material and Methods

### Patients and Tissue Specimens

We selected 35 MEC samples from patients treated at the A.C.Camargo Cancer Center and Barretos Cancer Hospital, Barretos, São Paulo, Brazil. Two experienced pathologists (FPS and VCA) in salivary gland tumors reviewed the diagnosis of all tumor cases and graded according to Auclair et al., 1992 (4). Demographic, clinical, pathological, therapeutic, and follow-up data were obtained from the patients’ medical records (**Table 1**). A reference RNA (Human Universal Reference Total RNA, Clontech, Mountain View, California, USA) was used and hybridized with both tumor RNA and normal salivary gland RNA. Six surrounding normal salivary glands (NSG/control) tissues were removed during surgical procedures of six MEC patients, and they were hybridized with reference RNA to further compare their mRNA and miRNA expressions with MEC’s (tumor) mRNA and miRNA expressions. All samples were collected from treatment-naive patients. Written informed consent was obtained from all patients before the sample collection. The National Human Research Ethics Committee approved the study (Protocol #1.380.762/2015).

**Table 1:** Demographic, clinical histopathological, therapeutic and follow-up findings of 35 mucoepidermoid carcinomas patients evaluated by mRNA and miRNA expression analyses.

Characteristics	Number of patients	
	miRNA analysis	mRNA analysis
Age (mean ± SD)	48.7 ± 19.7	47.7 ± 19.8
Gender		
Female	12	20
Male	13	14
Race		
Caucasian	17	26
Asian	1	1
NA	7	7
Anatomical site		
Parotid gland	13	16
Intra oral minor salivary gland and others*	7	8
Hard/soft palate	2	4
Tongue	2	4
Submandibular gland	1	2
cT stage		
T1-T2	8	10
T3-T4	10	14
NA	7	10
cN stage		
N0	13	18
N1	1	2
N2	4	4
N3	0	0
NA	7	10
cM stage		
M0	17	21
M1	1	3
NA	7	10
Tumor Grade		
Low	14	19
Intermediate	6	7
High	5	8
Vital status		
Alive	15	21
Deceased (cause of death MEC)	8	10
NA or dead of other causes#	2	3
Local recurrence		
Yes	6	6
No	18	27
NA	1	1
Treatment		
Surgery	8	13
Surgery and Radiotherapy	15	19
None	2, one received palliative RT	2, one received palliative RT
Distant Metastasis		
Yes	3	5
No	21	28
NA	1	1
Follow-up: median months (IQ range)	49.0 (62.0)	49.5 (59.8)

NA, Information not available; SD, standard deviation; IQ, Interquartile. *gingiva, maxillary sinus, eye, nasal fossa, nasal septum.

### miRNA Expression Analysis

miRNA expression analyses were performed in 25 out of the 35 fresh-frozen MEC samples and six NSG; no tissue or total RNA was available for analyses in the remaining 10 samples (**Table 1**). Hybridizations were performed using a one-color SurePrint 8X60K Human miRNA platform (G4870A, Agilent Technologies, Santa Clara, CA, USA), as recommended by the supplier. Background correction, quantile normalization, log2 transformation, and statistical tests were conducted using BRB ArrayTools software v. 4.4.0 (Biometric Research Branch, National Cancer Institute, Bethesda, MD, USA - https://brb.nci.nih.gov/BRB-ArrayTools/index.html). Sequences with more than 10% of MEC and NSG samples presenting undetectable expression (below background signal) were removed. The mean of the probes representing the same miRNA was used in the subsequent steps. miRNAs DE between MEC and NSG groups were identified with a p-value <0.05 (random variance t-test), false discovery rate (FDR) <0.05, and fold change (FC) ≥ 2 and ≤ -2. Supervised hierarchical clustering analysis was performed using 1-minus correlation distance and complete linkage (BRB array tools). Robustness of hierarchical clustering analyses was confirmed using pvclust package (R program) (**Supplementary Figure S1**). Data were deposited in the Gene Expression Omnibus (GEO) database with the accession number GSE199692.

### Gene Expression Analysis

Array-based gene expression analysis was performed in 34 out of the 35 fresh-frozen MEC samples and five NSG; one MEC and one NGS sample were excluded based on inferior RNA quality (**Table 1**). Hybridizations were performed using Two-color SurePrint G3 Human Gene Expression Microarray 8x60K (G4851B, Agilent) platform, as previously described (27). Data processing and analyses were carried out using similar parameters described for miRNA profiling (BRB array tools). Identification of DE mRNAs (p-value < 0.001, FDR < 0.05, FC ≥ 2 and ≤ -2) and supervised hierarchical clustering analysis were performed as described above. Pvclust package (R program) was used to confirm the robustness of hierarchical clustering analyses (**Supplementary Figure S1**). The data were deposited in the GEO database (accession number GSE169754).

### miRNA-mRNA Integrative Analysis

Target transcripts from the disrupted miRNAs were predicted using the miRWalk 2.0 tool (http://www.umm.uni-heidelberg.de/apps/zmf/mirwalk/), considering only the interactions predicted by at least three of four different bioinformatic algorithms (miRWalk, miRanda, RNAhybrid, and Targetscan). miRNA and mRNA expression data from 24 MEC samples tested by both procedures were integrated based on a significant negative correlation (Pearson correlation, p-value < 0.05) between predicted miRNA-mRNA interactions. Experimentally validated interactions were additionally obtained from the miRTarBase database (28).

### Pathway Enrichment Analysis

Pathway enrichment analysis was performed with KOBAS 3.0 (http://kobas.cbi.pku.edu.cn) and pathDIP (http://ophid.utoronto.ca/pathDIP) tools, comprising PANTHER, Reactome, and KEGG databases. Default parameters were adopted in KOBAS 3.0, and only experimentally detected protein-protein interactions were considered in PathDIP. The threshold used in both *in silico* tools was defined as p-value < 0.001 (hypergeometric test) and adjuscted p-value < 0.05 (Benjamini and Hochberg method).

### Cell Line Culture

Human Mucoepidermoid Carcinoma (UM-HMC-2) cells were isolated from the intermediate grade (stage IVb) parotid gland MEC of a 59-year-old Caucasian female and cultured according to Warner et al. (29).

### CRISPR/Cas9-Mediated Knockout of miRNA-22 and miR-205

miRNA-22 and miR-205 expression in UM-HMC-2 was confirmed using qRT-PCR (data not shown). Then, UM-HMC-2 cells were transfected with pSpCas9(BB)-2A-GF (PX458 expression vector, Addgene plasmid # 48138) expressing CRISPR-Cas9 and sgRNA targeting either miR-22 or miR-205 using Fugene HD transfection reagent (Promega, Madison, WI, USA). This resulted in transient expression of Cas9-sgRNA. Cells transfected with an empty plasmid were used as a control. After 72 hours, cells were sorted for GFP (Green fluorescent protein) positive population using a Sony SH800 cell sorter (Sony Biotechnology, San Jose, CA, USA), and were cloned as single cells per well in a flat bottom 96-well plate. Successfully expanded clones were then screened by capillary sequencing to detect nonhomologous end-joining CRISPR-Cas9 induced gene editing. Clones with predicted out of frame insertions and deletions (indels) were selected and expanded. The predicted effect of the CRISPR editing on miRNAs was assessed using the TIDE tool (30). Details of all sgRNAs and primers used in the experiments, as well as the CRISPR knockout efficiency, are summarized in the supplementary information (**Supplementary Figure S2A** and **Supplementary Table S1**).

### qRT-PCR for miRNA

In addition to sequencing, CRISPR knockout of miR-22 and miR-205 was confirmed using qRT-PCR. The miRNA was extracted with miRNeasy Tissue/Cell Advanced Mini Kit (Qiagen, Hilden, Germany) and transcripted to cDNA using miScript II RT Kit (Qiagen) following manufacturer’s instructions. The miScript universal primer and miRNA-specific primers for Hsa-miR-22-3p (MS00003220) and hsa-miR-205-5p (MS00003780) were purchased from Qiagen. The relative quantitative expressions were normalized to the endogenous control human RNU-6 (MS00033740) purchased also from Qiagen. Quantitative real-time PCR was performed on Applied Biosystems QuantStudio 5 Real-Time PCR System. qRT-PCR results are summarized in the supplementary information (**Supplementary Figure S2B**).

### qRT-PCR for mRNA

In order to study the effect of miR-knockouts, 11 genes were selected and evaluated by qRT-PCR: *PTEN, LAMC1, CADM1, HER3, MYCBP, SNAI1, YAP1, CD147, SMAD4, ESR1* (*ESR1*) and *ZEB2*. One thousand ng of the total RNA was used for cDNA synthesis. Synthesis was done using iScript cDNA Synthesis Kit (Bio-Rad Laboratories, Hercules, CA, USA) according to the manufacturer’s instructions. Two nanograms of cDNA was used for performing qRT-PCR with the Fast SYBR Green Master Mix (Thermo Fisher Scientific) as per the manufacturer’s instructions. The relative quantitative expression was normalized to the endogenous control *GAPDH*. The primers were purchased from Metabion (Planegg, Germany) and the sequences are summarized in the supplementary information (**Supplementary Table S1**). Quantitative real-time PCR was performed on Applied Biosystems QuantStudio 5 Real-Time PCR System.

### Cell Viability Assay

A CellTiter-Glo (CTG) 2.0 Luminescent Cell Viability Assay (Promega, Madison, WI, USA) was used to determine the effect of miR-22 and miR-205 on the cells’ viability. Briefly, 100 μL of cell suspension was dispensed in the Perkin Elmer ViewPlate-96 microplate with a clear flat bottom and black well walls (Perkin Elmer Inc., Waltham, MA, USA) for a final concentration of 1000 cells per well. After 72 hours, 100 μL of the CellTiter-Glo reagent was dispensed into the wells, and the luminescence reads were measured using a PHERAstar plate reader (BMG Labtech, Ortenberg, Germany).

### Scratch Wound Cell Migration and Invasion Assays

IncuCyte 96-well ImageLock Microplate wells (Sartorius, Göttingen, Germany) were coated with 300 μg/mL Myogel for migration and invasion assays (31). The cells were seeded at a density of 25,000 cells per well in 100 μL of complete medium for both assays. After 24 hours at 37°C, a 96-pin IncuCyte WoundMaker Tool (Sartorius) was used to make uniform wounds on the confluent monolayer of the cell. The wells were washed two times with media, and 100 μL of complete medium was added. For the invasion plate, 50 μL of Myogel-collagen gel (2.4 mg/mL Myogel, 0.8 mg/mL type I rat tail collagen) (Corning Incorporated, Corning, NY, USA) was added on top of the cells. After the gel was solidified, 50 μL of media was added, and the plates were transferred to an incubator. The wound closing was monitored automatically every 2 hours for two days using IncuCyte S3 Live-Cell Imaging System (Sartorius). Analysis of wound closing (width of the wound) was performed using Matlab. Mathematical function decorrelation was used to make the cells’ intensity substantially higher than the background.

### Spheroid Invasion Assay

The spheroid invasion assay was done according to Naakka et al. (32). The UM-HMC-2 cells were seeded at a concentration of 1000 cells per well in 50 µL of the complete medium using a U-shaped ultra-low attachment 96-well plate (Corning, New York, USA) and incubated for four days. Next, the spheroids were embedded in 50 μL Myogel-fibrin gel containing 0.5 mg/mL Myogel, 0.3 U/mL thrombin (Sigma-Aldrich), 33.3 mg/mL aprotinin (Sigma-Aldrich), and 0.5 mg/mL fibrinogen (Merck). After the Myogel-fibrin matrix (30 min) solidification, 100 μL of complete medium was added to the wells.

Images of the spheroids were captured daily using Nikon Eclipse TS100 Inverted Microscope (Nikon, Minato, Tokyo, Japan) at 4x magnification. Analysis of the spheroid invasion area and length of the longest branch was performed using ilastik (freeware) and Fiji ImageJ 1.51 software (33).

### Statistical Analysis

All *in vitro* assays were repeated at least three times, each performed at least in triplicate. Statistical analyses were carried out with SPSS v.25.0 (IBM Corporation, Chicago, IL, USA) and the GraphPad Prism software (v. 6.0; GraphPad Software Inc., La Jolla, CA, USA). Student’s T-test or One-way ANOVA followed by Bonferroni correction was used in posthoc analysis. A p-value < 0.05 was considered as statistically significant. Figures were created with Origin 2018b graphing software (OriginLab Corporation, Northampton, MA, USA).

Overall survival analysis was performed using the Kaplan-Meier estimator with the log-rank test in IBM SPSS Statistics for Windows, Version 25.0 (IBM Corp. Armonk, NY, USA). The miRNA expression values were dichotomized below and above the median (p-value < 0.05). A random variance t-test using BRB ArrayTools software (v. 4.4.0) was applied to investigate differences in the miRNA expression in relation to the histological grade, lymph node, and distant metastasis (p-value < 0.05, FDR < 0.05).

## Results

**Figure 1:**
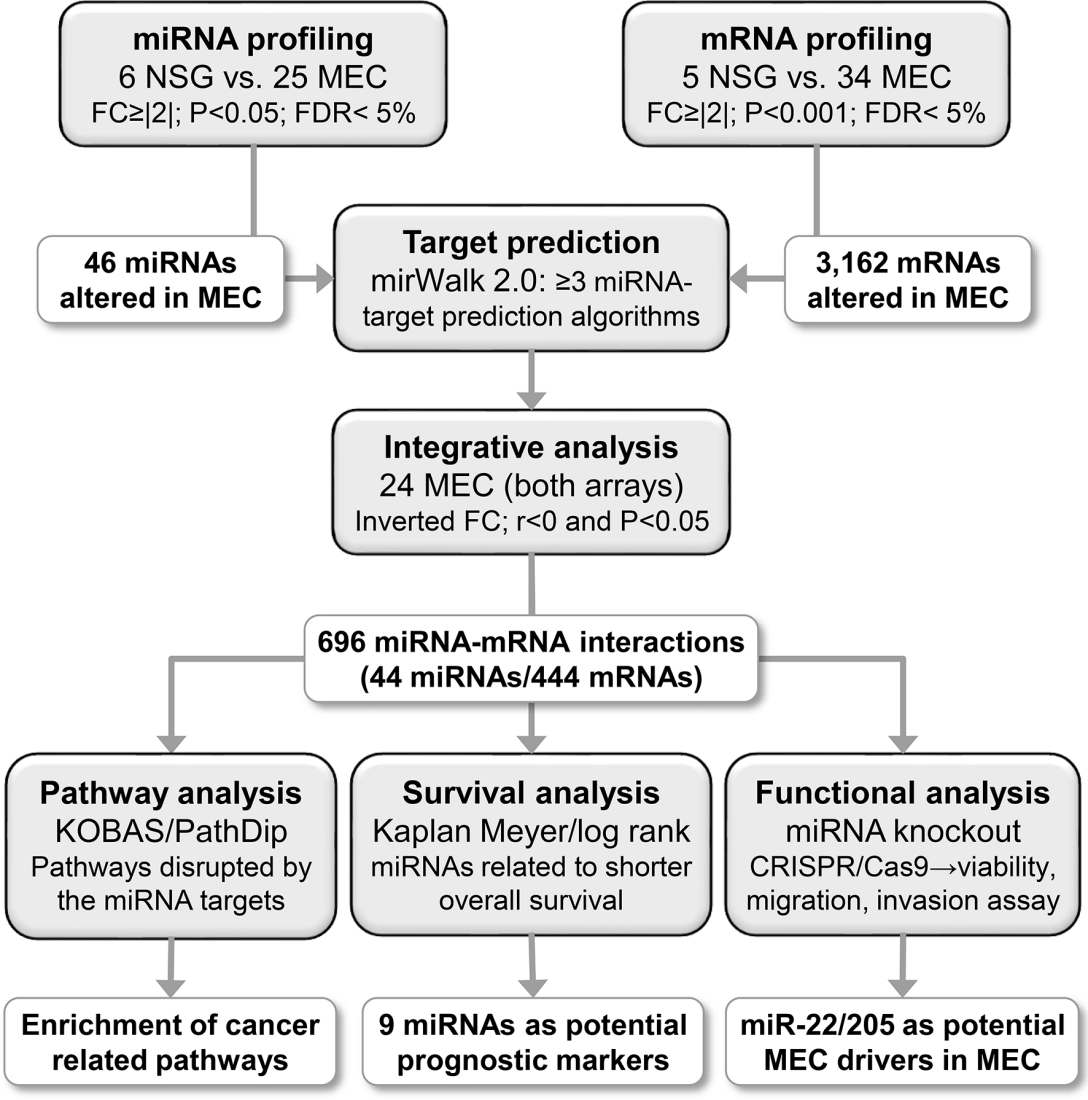
Study design and main results obtained from the miRNA and mRNA expression analyses. First, a miRNA and mRNA global expression analyses revealed 46 miRNAs and 3,162 mRNAs differentially expressed in MEC compared to SNG. An integrative analysis was carried out using predicted miRNA-mRNA interactions, generating a network containing 44 miRNAs and 444 mRNAs (696 interactions). The target genes were associated with cancer-related pathways, and nine miRNAs were associated with shorter overall survival. A knockout assay was performed for miR-22 and miR-205 (CRISPR/Cas9), resulting in viability, migration, and invasion reduction, which indicate their role as putative cancer drivers in MEC.

The mean age of whole patient group was 46.9 ± 20.2 years (range 12 to 82 years old). Female patients were more frequently affected by MEC than males (ratio 1.4:1). Parotid was the most common anatomical site, followed by minor salivary glands of the palate and other sites. Twenty cases presented with low histologic grade, seven with intermediate-grade, and nine with high-grade at diagnosis. T3-T4 tumors at diagnosis were found in 14 cases. Six patients presented lymph node involvement at diagnosis and three patients presented distant metastases at diagnosis. Twenty patients were treated with surgery and radiotherapy, while 14 received surgery only. Follow-up time ranged from 4 to 188 months (median 49,5 months). Demographic, clinical, histopathological, therapeutic and follow-up features are detailed in **Table 1**. The study design, methodologies, and the foremost results are presented in **Figure 1**.

### miRNA and mRNA Expression Profile of MEC

**Figure 2:**
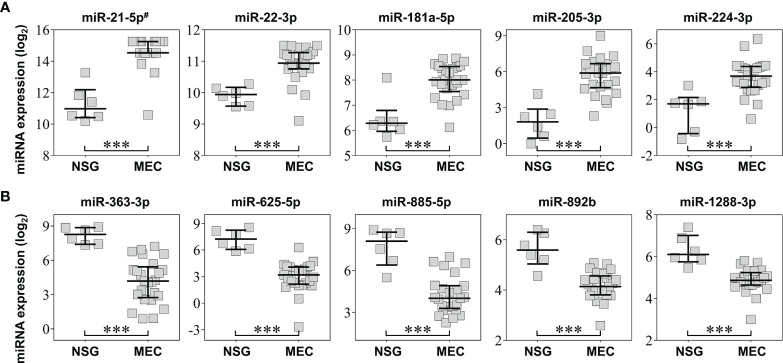
Top five most significant overexpressed **(A)** and underexpressed **(B)** miRNAs obtained in the microarray analysis. The error bars and middle line represent the interquartile range and median, respectively. NSG: surrounding normal salivary gland tissues; MEC: mucoepidermoid carcinoma tissues. ^#^miR-21-3p was omitted (both mature sequence from miR-21 precursor were highly significant). ***P < 0.001 (t test).

After excluding uniformly low expressed miRNAs in MEC and NSG samples, 530 miRNAs and 19,911 mRNAs were considered for further analysis. We found 46 DE miRNAs (18 overexpressed and 28 underexpressed) in MEC (**Supplementary Table S2**). The most significant (P adjusted ≤ 0.005) overexpressed miRNAs included miR-21-5p (FC=10.2), miR-22-3p (FC=2.0), miR-181a-5p (FC=2.9), miR-205-3p (FC=14.7), and miR-224-3p (FC=5.3). The miR-363-3p (FC =-16.3), miR-625-5p (FC =-18.5), miR-885-5p (FC =-10.7), miR-892b (FC =-2.7), and miR-1288-3p (FC =-2.7) were significantly underexpressed (**Figure 2**). A similar approach used for mRNAs unveiled 3,162 mRNAs differentially expressed in MEC (1,488 overexpressed and 1,674 underexpressed (**Supplementary Table S3**).

**Figure 3:**
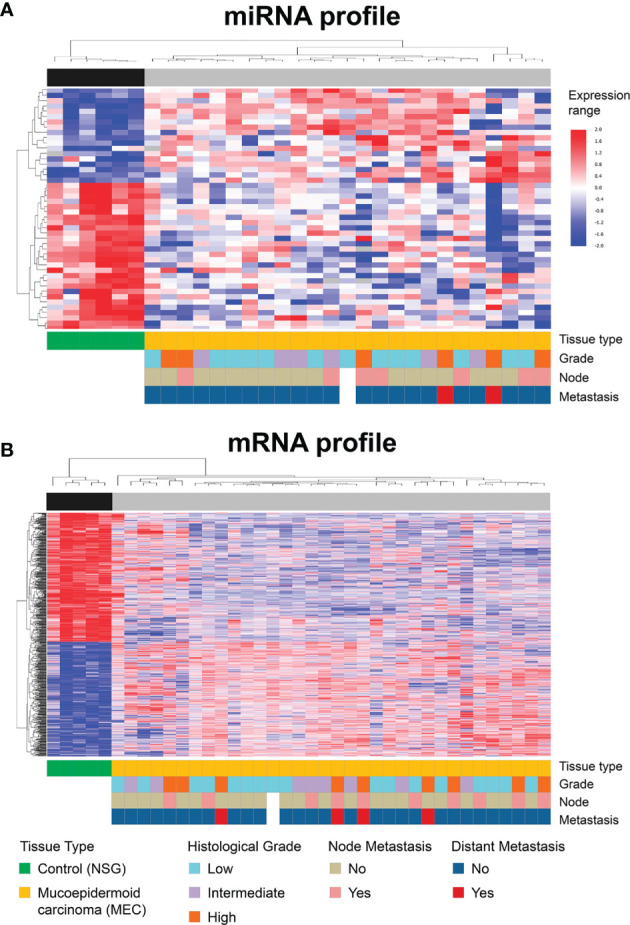
Supervised hierarchical clustering analysis considering the miRNA **(A)** and mRNA **(B)** expression profiles. The dendrograms show a complete separation between MEC and NSG samples according to the 47 miRNAs **(A)** and 3,162 mRNA differentially expressed **(B)**. The samples are represented in columns and miRNAs/genes in rows.

Supervised hierarchical clustering analysis based on the DE transcripts revealed two clusters in both miRNA (**Figure 3A**) and mRNA (**Figure 3B**). Although these two main clusters completely separated MEC from NSG samples, no association was observed when comparing the clinical-pathological parameters (histological grade, lymph node involvement, and distant metastasis) with the clusters generated by both miRNA and mRNA analysis.

The miRNA-target prediction analysis resulted in 20,816 miRNA-mRNA putative interactions. The integrative analysis revealed a miRNA-mRNA network comprising 696 negatively correlated interactions and inverted FCs (44 miRNAs and 444 mRNAs) (**Supplementary Table S4**). The main biological pathways uncovered by miRNA targets corroborated by the integrative analysis were cell signaling, cell cycle, and cancer-related pathways (**Table 2**).

**Table 2:** Biological pathways enriched (P value < 0.001 and P adjusted < 0.05) by the genes detected in the miRNA-mRNA integrative analysis (KOBAS 3.0 and Pathdip *in silico* pathway tools).

Biological Pathways	Database	KOBAS 3.0		Pathdip*	
		P value	P adj	P value	P adj
Signal Transduction	Reactome	4E-12	4E-09	6E-05	8E-03
Post-translational protein modification	Reactome	1E-08	4E-06	2E-04	1E-02
Membrane Trafficking	Reactome	2E-07	2E-05	5E-04	2E-02
Diseases of signal transduction	Reactome	3E-06	2E-04	9E-05	8E-03
Signaling by Rho GTPases	Reactome	8E-06	4E-04	1E-04	1E-02
EPH-Ephrin signaling	Reactome	2E-05	8E-04	9E-05	8E-03
Cell Cycle	Reactome	2E-05	9E-04	4E-05	9E-03
RHO GTPase Effectors	Reactome	3E-05	1E-03	8E-06	3E-03
Proteoglycans in cancer	KEGG	2E-04	4E-03	5E-05	8E-03
Cell Cycle, Mitotic	Reactome	2E-04	4E-03	2E-04	1E-02
DNA Double-Strand Break Repair	Reactome	2E-04	5E-03	1E-03	3E-02
EPH-ephrin mediated repulsion of cells	Reactome	4E-04	8E-03	6E-04	2E-02
MicroRNAs in cancer	KEGG	9E-04	1E-02	6E-05	7E-03

KEGG, Kyoto Encyclopedia of Genes and Genomes; *Experimentally detected protein-protein interactions.

**Figure 4:**
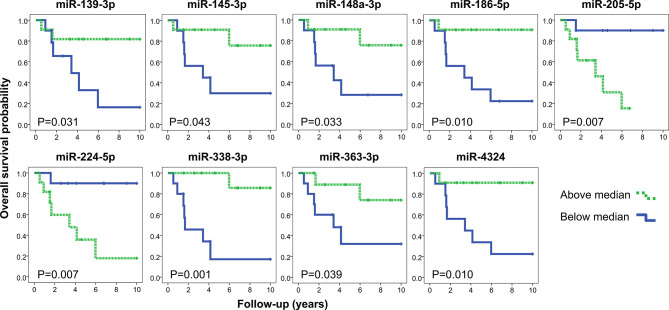
Kaplan-Meier representation of overall survival according to the expression levels of nine miRNAs (log rank test P<0.05). The quantifications obtained by the microarray analysis were stratified in below (blue) and above (green) the median values. Note: miR-224-3p was omitted (both mature sequences from mir-224 precursor were associated with overall survival).

Lower expression levels of miR-582-5p, miR-3125, and miR-4324 were found in high-grade MEC compared to low and intermediate grades (**Supplementary Figure S3**). Increased expression levels of miR-205-5p and miR-224-5p (both overexpressed in MEC) and decreased expression levels of miR-139-3p, miR-145-3p, miR-148a-3p, miR-186-5p, miR-338-3p, miR-363-3p and miR-4324 were significantly related to worse overall survival in MEC patients (**Figure 4**).

Among the list of differentially expressed miRNAs, we selected miR-22 and miR-205 for functional assays for the following reasons: they were significantly overexpressed (adjuscted p-value <0.005) (**Figure 2**), presented high interactivity in the integrative analysis (>10 underexpressed mRNA predicted targets negatively correlated with the miRNA expression) (**Supplementary Table S4**), and showed clinical association with worse prognosis (increased miR-205 expression was associated with shorter overall survival) (**Figure 4**).

### Knockout of miR-205 Decreases MEC Cell Viability While the Knockout of miR-22 Reduces MEC Cell Migration and Invasion

**Figure 5:**
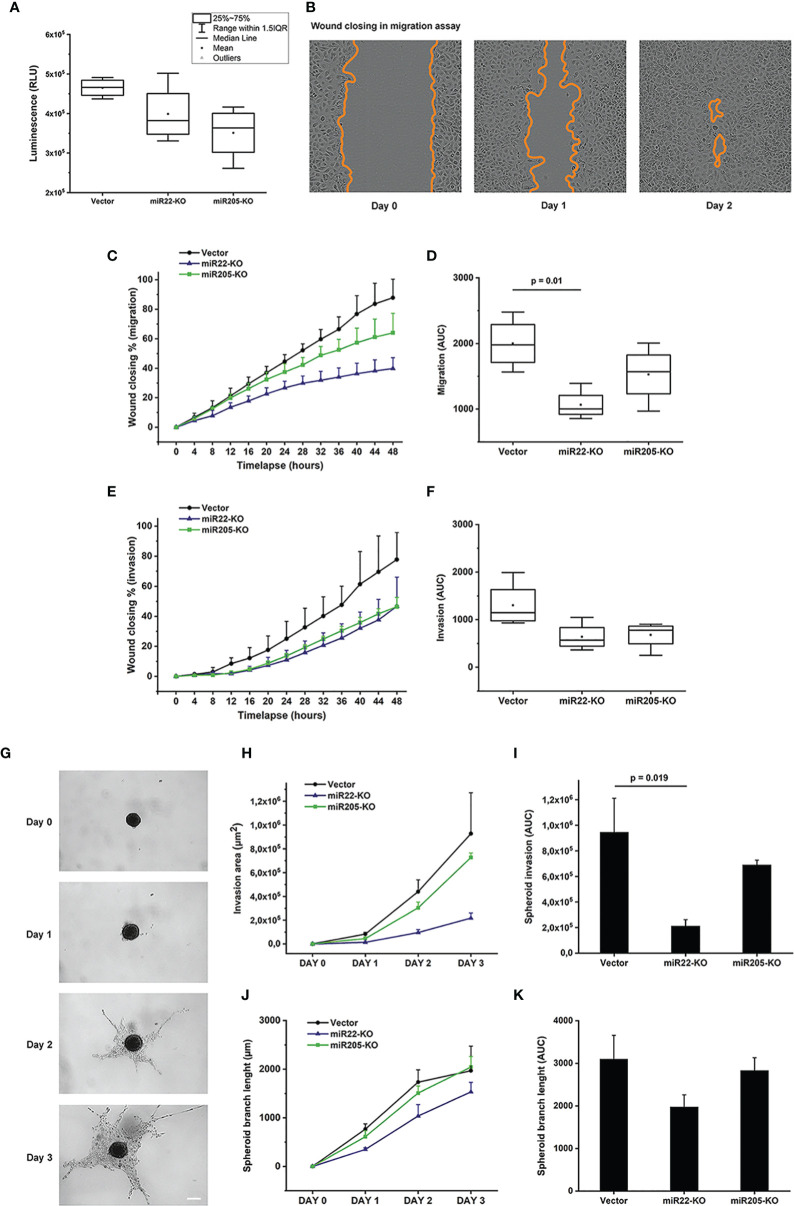
Cell viability, migration and invasion assays performed using the UM-HMC-2 cell line. **(A)** UM-HMC-2 cells were cultured for three days and the cell viability was measured using luminescent cell viability assay. Although not statistically significant, the cell viability was decreased in both miR22- and miR205-knockout cell lines compared to the cell line transfected with an empty plasmid vector. **(B–D)** UM-HMC-2 cells were cultured on Myogel matrix and cell migration was evaluated using scratch wound cell migration assay. **(B)** Representative image of migration distance at 0, 24, and 48 hours after wounding. **(C, D)** Quantification of cell migration in scratch wound assay. miR22- and miR205-knockout cell lines migrated slower than the vector cell line. Statistically significant difference was denoted between vector and miR22-KO cell lines. **(E, F)** UM-HMC-2 cell invasion through Myogel-collagen in scratch wound cell invasion assay. UM-HMC-2 cells were cultured in Myogel-collagen matrix, and cell invasion was evaluated using scratch wound cell invasion assay. miR22- and miR205-knockout cell lines invaded slower than vector cell line (p-value > 0.05). **(G–K)** UM-HMC-2 cell invasion through Myogel-fibrin in spheroid invasion assay. Cells were cultured in U-shaped ultra-low attachment 96-well plate wells and embedded in Myogel-fibrin matrix. Spheroids were observed under a light microscope and the invasion area and the spheroid branch length were analyzed using ilastik and ImageJ software. **(G)** Representative images of spheroid invasion at different time points. Scale bar = 200 μm (Original magnification X4). **(H, I)** Quantification of cell invasion in 3D spheroid invasion assay. Knockout of miR22 and miR205 reduced tumor cell invasion. Difference between vector and miR22-KO cell lines reached statistical significance. **(J, K)** Quantification of spheroid branch length revealed that miR22- and miR205-knockout cell line spheroids did not extend as far as vector cell line (p-value > 0.05). Data are presented as means ± SD of 3-4 independent experiments, each at least in triplicate. p < 0.05 is considered as significantly different compared to vector control.

We explored the use of the CRISPR-Cas9-based method to knockout miR-22 and miR-205 in MEC. Cell viability, migration, and invasion assays were performed in the MEC cell line UM-HMC-2. The cell viability was the lowest in the miR-205 knockout, followed by miR-22-knockout cells, but with no statistical significance (**Figure 5A**).

The scratch wound migration assay showed that miR-22 and miR-205 knockouts reduced cell migration. The effect was the same in both knockout cell lines compared to the empty vector, but miR-22-knockout cells migrated significantly slower than the control cells **(****Figures 5B–D** and **Supplementary Figure S4A**). The cell lines showed different invasion speeds, and the knockouts invaded slower in both scratch wound invasion (**Figures 5E–F** and **Supplementary Figure S4A**) and spheroid invasion assays (**Figures 5G–K** and **Supplementary Figure S4B**). Both miR-22 and miR-205 knockout cell lines invaded slower than the cell line with empty gRNA vectors. However, the effect was statistically significant only when miR-22-knockout cells were compared to the empty vector in the spheroid invasion assay.

### Knockout of miR-22 Induces ESR1 and Knockout of miR-205 Induces ZEB2 Expression

**Figure 6:**
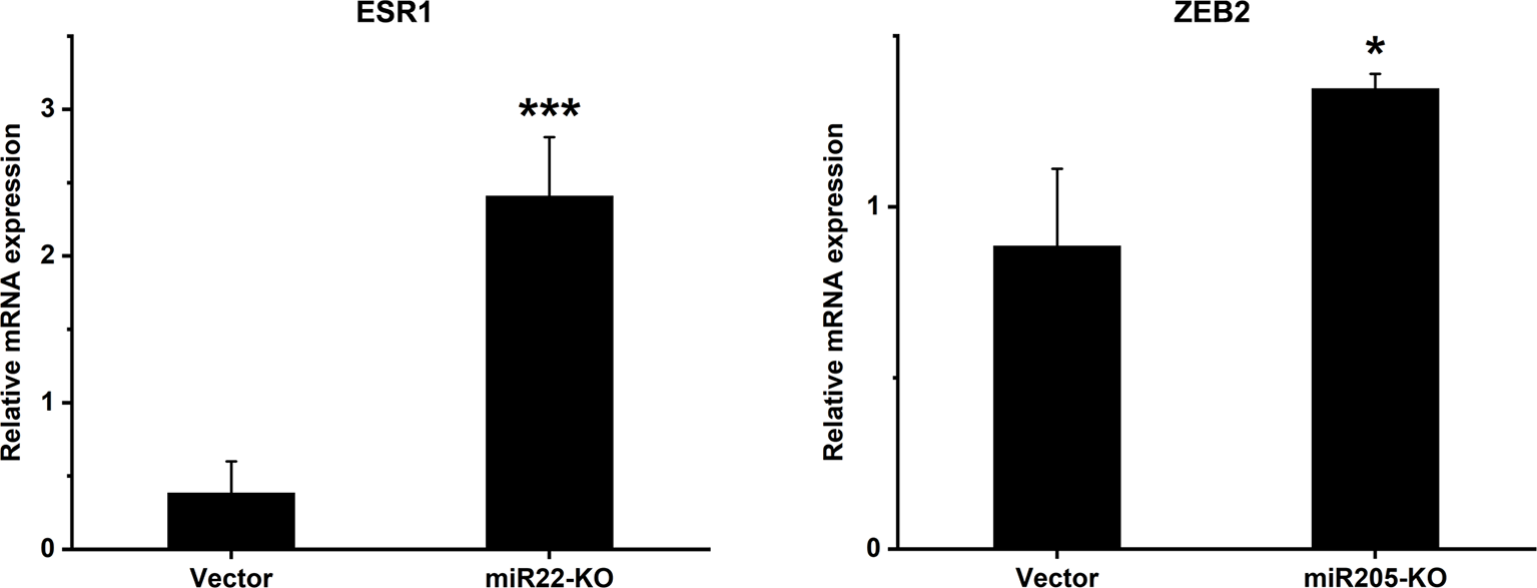
mRNA expression levels of selected genes after miR-knockout. Expression levels of *ESR1* in miR22-KO and *ZEB2* in miR205-KO cell lines were analysed using qRT-PCR. The relative mRNA levels are shown after normalization to GAPDH. *ESR1*, Estrogen receptor alpha; *ZEB2*, Zinc finger E-box binding homeobox 2. Data are presented as means ± SD. *p ≤ 0.05, *** ≤ 0.001.

In order to understand the mechanism behind the effect of miR-22 and miR-205 knockout on MEC cell behaviour, we studied the expression of specific molecules: *PTEN, LAMC1, CADM1, HER3, MYCBP, SNAI1, YAP1, CD147, SMAD4, ESR1* and *ZEB2* which, based on the literature, are known to be targets either for miR-22 or miR-205. We reported significant differences in two of the targets: estrogen receptor alpha (*ESR1*) for miR-22 and zinc finger E-box-binding homeobox 2 (*ZEB2*) and miR-205 (**Figure 6**). These molecules influence cell proliferation, migration and invasion (34–36). As expected, miR-22 knockout cells have significantly higher expression of *ESR1*, and miR-205 knockout cells have significantly higher expression of *ZEB2* compared with the empty vector.

## Discussion

Varied clinical behavior and multiple histologic grading systems have challenged pathologists in prognostication of MEC and clinicians in making an appropriate treatment decision for the patients (37). Moreover, the differential diagnosis between a salivary gland MEC and other lesions, such as salivary duct cyst, cystadenoma, or glandular odontogenic cyst may be difficult in some situations. In particular, small incisional biopsies are often problematic in the diagnostic workup. The presence of the *CRTC1-MAML2* fusion gene can be helpful for the diagnosis of MEC, but it is not found in all cases of MEC, and there is contradiction about some benign conditions (38–40). Mucoepidermoid carcinomas of the salivary gland are poorly explored at the molecular level. Therefore, genetic studies can unravel diagnostic, prognostic, and predictive markers, as reported in several tumor types.

In the present study using large-scale expression analyses, we found 46 miRNAs and 3,162 mRNAs differentially expressed compared to normal salivary glands. In agreement with our present miRNA findings, a previous MEC study reported that miRNA-205 and miRNA-22 were amongst the highest overexpressed miRNAs in MEC, while miRNA-885-5p and miRNA-375 were downregulated (26).

Two earlier studies have investigated global gene expression in MEC (22, 23), but none of the genes reported were found in our analysis. A possible explanation for this discordance may be the small number of MEC cases (2 and 6) investigated in the earlier studies and/or the different methodological strategies. For instance, Leivo et al. (22) focused on comparing different histological types of salivary gland malignancies, which might explain the disparities compared with our findings.

Although we could not investigate the *CRTC1-MAML2* status in our sample set due to a lack of sample material, we observed a decreased *CRTC1* expression level. In MEC, the *CRTC1-MAML2* gene fusion activates *CREB/Cyclic* AMP related genes and possibly the Notch pathway (11, 41–45). Recently, Chen et al. (2021) (46) suggested that deregulated *p16-CDK4/6-RB* signaling is a cooperating event in the progression of MEC with the *CRTC1-MAML2* fusion. The authors also suggested that *EGFR* and *CDK4/6* inhibitors are potentially useful to treat MEC patients.

An integrative analysis was conducted to elucidate the role of miRNAs and their mRNA targets and the core genes and pathways involved in MEC. We found 669 miRNA-mRNA interactions (44 miRNAs and 444 mRNAs) involving cancer-related pathways such as miRNAs in cancer, cell cycle and signal transduction, ERK/MAPK signaling, EIF2 signaling, PI3K/AKT, among others. These findings provide supportive evidence for the detection of drivers involved in MEC pathogenesis. A set of these transcripts was associated with poor prognostic features, such as high histological grade. For instance, a decreased expression of miR-582-5p in MEC was related to high-grade tumors. Previously, miRNA-582-5p downregulation was described in salivary gland tumors (47, 48), and its induction inhibited invasion and migration in salivary adenoid cystic carcinoma (AdCC) (48). We found that the target of this miRNA, *EZH2*, was overexpressed and related to high-grade MEC (**Supplementary Table S4** and **Supplementary Figure S3**). *EZH2* is a member of the polycomb group of proteins involved with transcription regulation through chromatin remodeling (49). Increased EZH2 protein expression has been reported in MEC, myoepithelial carcinoma of salivary glands, and AdCC (50–52). In AdCC, increased EZH2 expression was associated with a worse prognosis.

Significantly decreased miR-4324 expression was detected in our high-grade MEC compared to low/intermediate-grade tumors, and it was also associated with shorter overall survival. miR-4324 has been shown to be underexpressed in a subset of *PTEN* deficient breast cancer patients with exceedingly poor prognoses (53). *PIK3CA* and *PTEN* inactivating mutations are frequent events in high-grade MEC (54). Interestingly, a highly predicted interaction between miR-205-3p and *PLAC8* from the PI3K pathway was observed in our integrative analysis. A recent study demonstrated that *PLAC8* contributes to cell proliferation and suppresses cell apoptosis in breast cancer by activating the PI3K/AKT/NF-κB pathway (55).

Based on established criteria, including increased expression levels, high interactivity in the integrative analysis, and association with clinical parameters, we selected two miRNAs, miR-205 and miR-22, for functional assays. These two miRNAs were among the highest overexpressed miRNAs in previously described MEC cases (26). miR-205 was one of the most significantly overexpressed miRNAs, and it was associated with shorter overall survival in our MEC cases. Overexpression of this miRNA has been reported in several cancers, including AdCC and head and neck squamous cell carcinomas (56–58). A previous study suggested that miR-205-5p targets *PTEN* to regulate the epithelial mesenchymal transition through the PI3K/AKT pathway (58).

Since miR-22 was one of the highest overexpressed miRNAs in MEC, it was selected for knockdown and functional experiments. Dysregulation of this miRNA has been reported in several tumor types (59) and implicated in the regulation of cell growth, cell cycle, apoptosis, and invasion (60, 61). *MYC* and *PI3K/AKT* can induce miR-22 gene expression, which in turn targets *PTEN* (62). Since PTEN is a repressor of AKT, miR-22 could act as a key element in a positive feedback of the *PI3K/AKT* pathway to cause downregulation of *PTEN* (59). As previously described in MEC (13), this miRNA also induces chromosomal instability (63). Knockdown of miR-22 showed a consistent reduction of viability, migration, and invasion of MEC cells. However, the effect on migration and invasion was stronger and seems not to be as a result of reduced viability which was only mild and not significant.

Previous studies have reported that miR-22 represses *ESR1* expression in breast cancer and lead to a reduction in estrogen signaling (34). In line with that, we showed that the miRNA-22 knockout increased *ESR1* expression levels. *ZEB2* was reported to negatively correlate with miR-205 levels in esophageal squamous cell carcinoma cells (35) and silencing of *ZEB2* lead to suppressed cell viability, migration, and invasion in laryngeal squamous cell carcinoma cells (36). Our data showed an upregulation of *ZEB2* in miR-205-knockout cells which is in line with the reports above. Additionally, *ZEB2* has been shown to directly bind to the E-cadherin promoter and repress its transcription (64). Loss of E-cadherin is one of the main initiation events of epithelial to mesenchymal transition (EMT) and thus plays an important role in cancer progression. The biological mechanism behind these actions remains to be elucidated in future studies.

## Conclusion

Although we investigated a limited number of cases, we described a transcriptomic profile distinguishing MEC from normal salivary glands. The integrative analysis highlighted miRNA-mRNA interactions, and cancer-related pathways were described. Comparison with other studies using similar strategies was limited due to the absence of available miRNA-mRNAs expression data in public databases. However, our list of differentially expressed miRNAs-mRNAs revealed that PTEN and PI3K/AKT pathways were altered in MEC. Our *in vitro* functional assays indicate that miR-22 and miR-205 deficiencies reduce cell viability, migration, and invasion in a MEC cell line by enhancing the expression of *ZEB2* and *ESR1* mRNAs. Taken together, our findings suggest that these dysregulated miRNAs have a pathogenic role in MEC.

## Data Availability Statement

The original contributions presented in the study are included in the article/**Supplementary Material**. Further inquiries can be directed to the corresponding author.

## Ethics Statement

The studies involving human participants were reviewed and approved by The National Human Research Ethics Committee (Protocol #1.380.762/2015). Written informed consent to participate in this study was provided by the participants’ legal guardian/next of kin.

## Author Contributions

Study concept and design: FP-S, TS, and SR. Data Acquisition: EN, MB-F, HK, KT, and AB. Quality control and data algorithms: MB-F, SA, FM, IS, and SL. Data analysis and interpretation: FP-S, EN, MB-F, and FM. Statistical analysis: MB-F, SA, FM, and IS. Manuscript preparation: FP-S and EN. Manuscript editing: EN, FP-S, TS, and SR. Manuscript review: CS-N, RC, LK, AM, VA, IL, TS, SR, and AA-S. All authors contributed to the article and approved the submitted version.

## Funding

This work was supported by grants from Fundação de Amparo à Pesquisa do Estado de São Paulo - FAPESP (2012/10382-5) and (2013/04045-9), and Doctoral Programme in Clinical Research (KLTO), Faculty of Medicine, University of Helsinki, Finland; Sigrid Jusélius Foundation; the Cancer Society of Finland, Jane and Aatos Erkko Foundation, and Helsinki University Central Hospital research funds. SR acknowledges support from Research Council Lillebaelt Hospital, Denmark.

## Conflict of Interest

The authors declare that the research was conducted in the absence of any commercial or financial relationships that could be construed as a potential conflict of interest.

## Publisher’s Note

All claims expressed in this article are solely those of the authors and do not necessarily represent those of their affiliated organizations, or those of the publisher, the editors and the reviewers. Any product that may be evaluated in this article, or claim that may be made by its manufacturer, is not guaranteed or endorsed by the publisher.

## Supplementary Material

Supplementary Figure S1 Bootstrap analysis to estimate the cluster stability. B = 1000 bootstraps conducted with pvclust package (R program).

Supplementary Figure S2 **(A)** CRISPR knockout efficiency and indel spectrum. The predicted effect of the CRISPR-editing on miRNAs was assessed using TIDE online tool by The Netherlands Cancer Institute, Amsterdam, Netherlands (https://tide.nki.nl/). **(B)** qRT-PCR assay reveals down-regulation of miR-22-3p (p < 0.008) and miR-205-5p (p < 0.004) expression in knockout cells compared to vector control.

Supplementary Figure S3 Differentially expressed miRNAs according to the histological grade. miR-582-5p (FC= -5.1; P=0.0001; FDR =0.0066), miR-4324 (FC= -3.7, P=0.0019, FDR=0.0305) and miR-3125 (FC=-2.1, P=0.0031, FDR=0.0305) were all underexpressed in high-grade mucoepidermoid salivary gland carcinoma compared to low/intermediate grade. ***P < 0.001, **P < 0.01 (t test).

Supplementary Figure S4 UM-HMC-2 cell migration and invasion assays. **(A)** Representative images of UM-HMC-2 cell migration and invasion distance at 0, 24, and 48 hours in wound scratch wound assay. **(B)** Representative images of UM-HMC-2 cell invasion through Myogel-fibrin in spheroid invasion assay at different time points. Scale bar = 200 μm (original magnification X4).

Supplementary Table S1 Primers used in this study. Oligonucleotide pairs for construction of gRNA expression plasmids, primer sequences used to amplify the target site before the Sanger sequencing and primers for target gene qRT-PCR (F, forward; R, reverse).

Supplementary Table S2 Differentially expressed miRNA in salivary gland mucoepidermoid carcinoma (MEC) compared to normal salivary gland (NSG) tissues.

Supplementary Table S3 Differentially expressed mRNAs in MEC compared to non-neoplastic salivary gland tissues (excel file).

Supplementary Table S4 MicroRNA and target-mRNA interactions retrieved from the integrative analysis, comprising the transcripts differentially expressed in MEC (excel file).
