# Supplementary figures and images for: miR-22 and miR-205 Drive Tumor Aggressiveness of Mucoepidermoid Carcinomas of Salivary Glands
Source: Front Oncol. 2022 Feb 9;11:786150. doi: 10.3389/fonc.2021.786150 (PMC8864291; doi:10.3389/fonc.2021.786150)

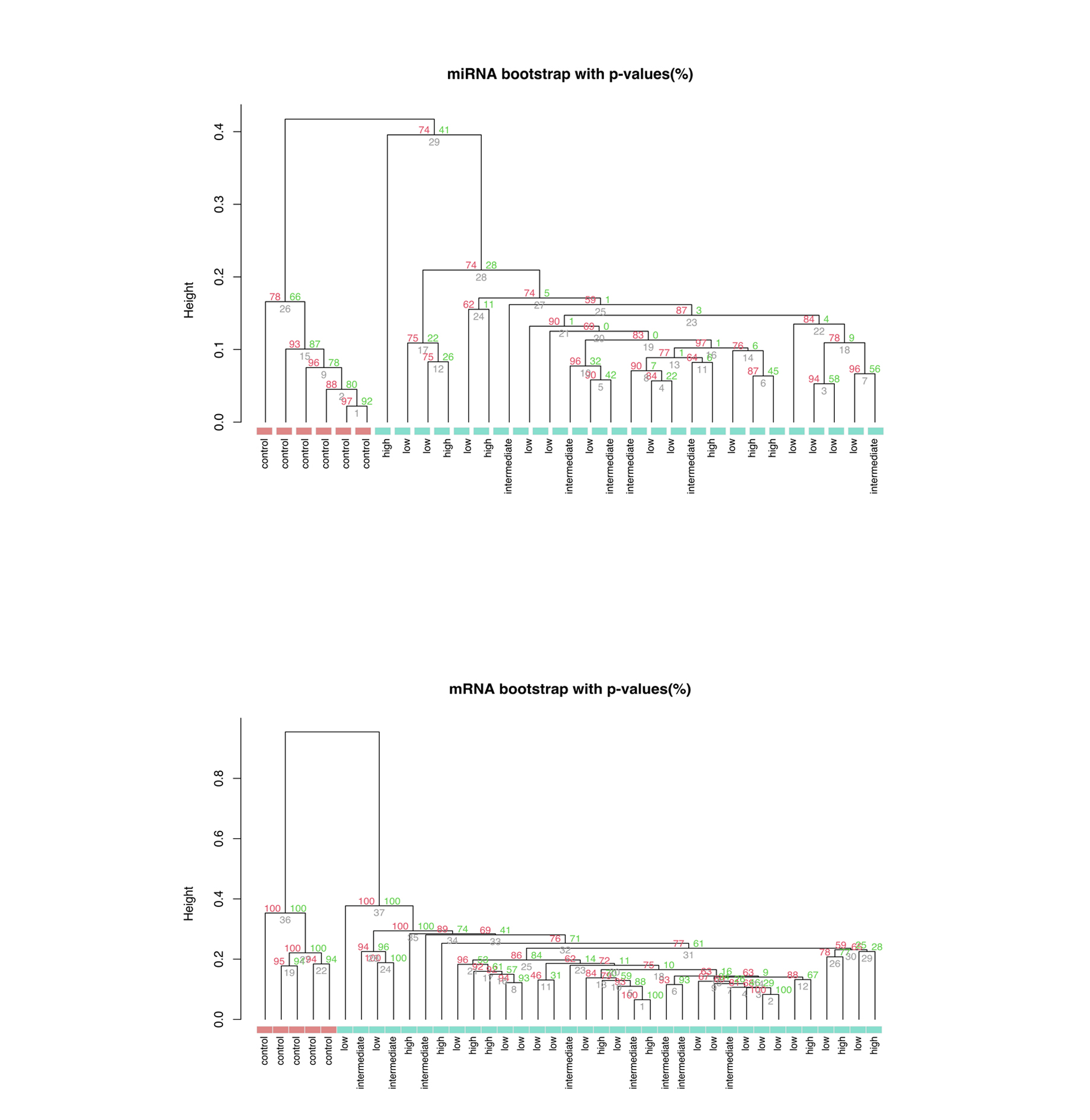

Supplement: Supplementary Figure S1 — Bootstrap analysis to estimate the cluster stability. B = 1000 bootstraps conducted with pvclust package (R program). [file Image_1.jpg]

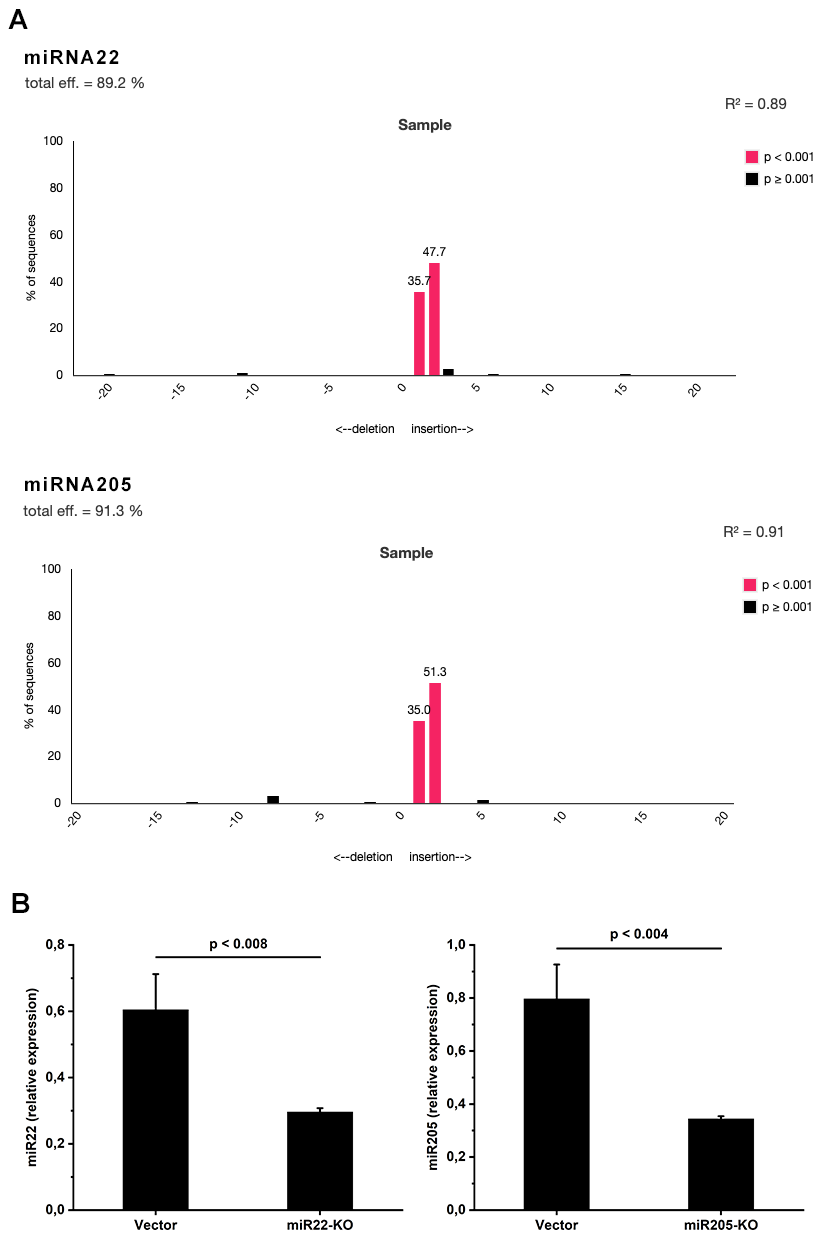

Supplement: Supplementary Figure S2 — (A) CRISPR knockout efficiency and indel spectrum. The predicted effect of the CRISPR-editing on miRNAs was assessed using TIDE online tool by The Netherlands Cancer Institute, Amsterdam, Netherlands (https://tide.nki.nl/). (B) qRT-PCR assay reveals down-regulation of miR-22-3p (p < 0.008) and miR-205-5p (p < 0.004) expression in knockout cells compared to vector control. [file Image_2.tif]

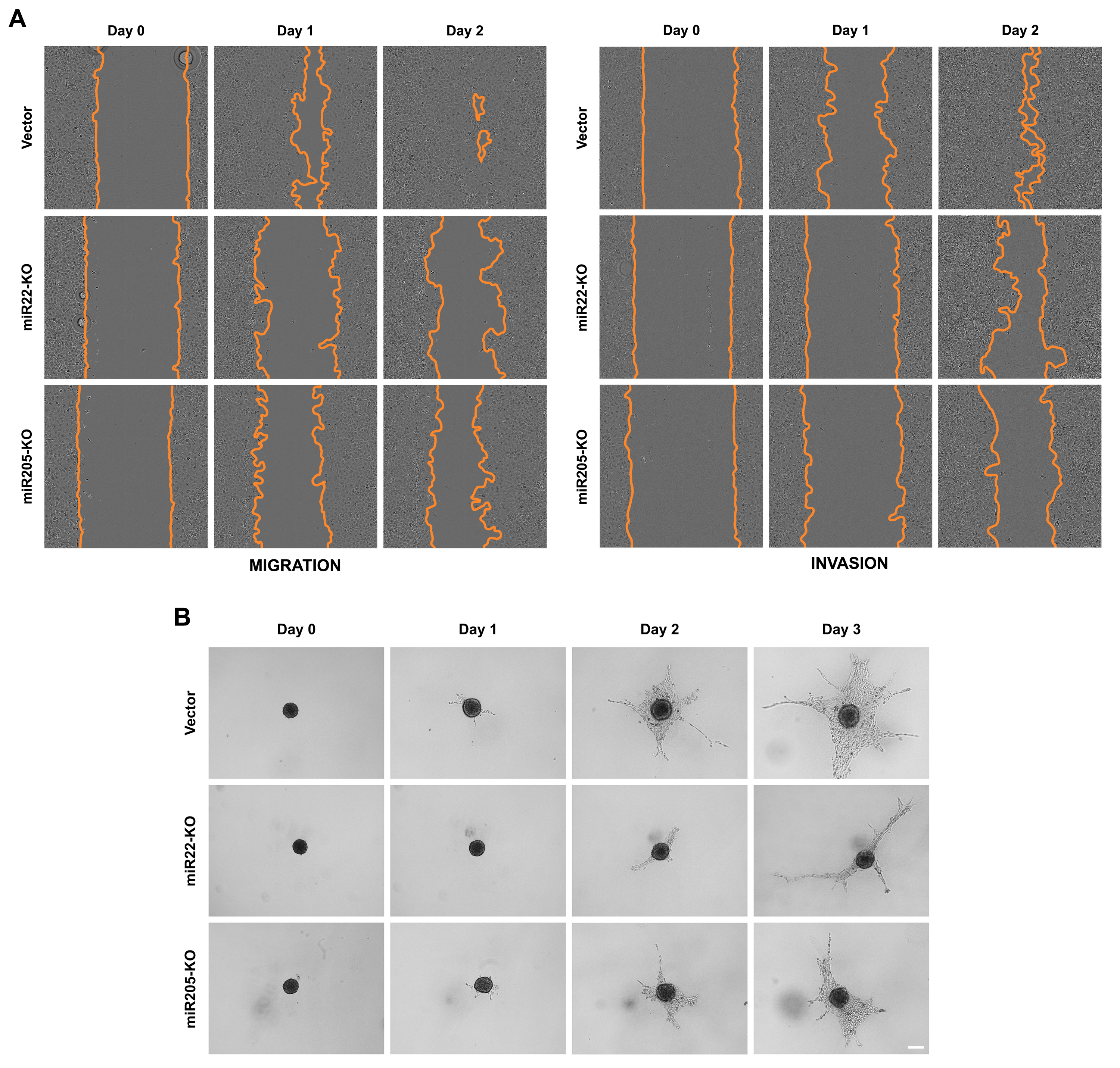

Supplement: Supplementary Figure S4 — UM-HMC-2 cell migration and invasion assays. (A) Representative images of UM-HMC-2 cell migration and invasion distance at 0, 24, and 48 hours in wound scratch wound assay. (B) Representative images of UM-HMC-2 cell invasion through Myogel-fibrin in spheroid invasion assay at different time points. Scale bar = 200 μm (original magnification X4). [file Image_4.jpg]
