# Supplementary material for: miR-22 and miR-205 Drive Tumor Aggressiveness of Mucoepidermoid Carcinomas of Salivary Glands
Source: Front Oncol. 2022 Feb 9;11:786150. doi: 10.3389/fonc.2021.786150 (PMC8864291; doi:10.3389/fonc.2021.786150)
Supplement: Supplementary Table S1 — Primers used in this study. Oligonucleotide pairs for construction of gRNA expression plasmids, primer sequences used to amplify the target site before the Sanger sequencing and primers for target gene qRT-PCR (F, forward; R, reverse). [file Table_1.docx]

**Supplementary Table S1.** Primers sets used in this study. Oligonucleotide pairs for construction of gRNA expression plasmids, primer sequences used to amplify the target site before the Sanger sequencing and primers for target genes qRT-PCR (F, forward; R, reverse).

| Supplementary Data. Primers used in this study | |
| --- | --- |
|  |  |
| Oligonucleotide pairs for construction of gRNA expression plasmids | |
| Name | **Sequence (5’-3’)** |
| MIR22-KO-F | CACCGAGCCGCAGTAGTTCTTCAG |
| MIR22-KO-R | AAACCTGAAGAACTACTGCGGCTC |
| MIR205-KO-F | CACCGATTTCAGTGGAGTGAAGTTC |
| MIR205-KO-R | AAACGAACTTCACTCCACTGAAATC |
|  |  |
| Primers for amplification of the target before Sanger sequencing | |
| Name | **Sequence (5’-3’)** |
| MIR22-F | GCAAAGGCTCTCCAACTTGC |
| MIR22-R | AGCGAGGTTAACAGCTTCCG |
| MIR205-F | CAGGTCCTTGACATCTCCCA |
| MIR205-R | TGATCACATTTCTCTCTGGCTGT |
|  |  |
| Primers for target gene qRT-PCR | |
| Name | **Sequence (5’-3’)** |
| *GAPDH-F* | AAGGTCATCCCTGAGCTG |
| *GAPDH-R* | TGCTGTAGCCAAATTCGTTG |
| *PTEN-F* | CCAGGACCAGAGGAAACCT |
| *PTEN-R* | GCTAGCCTCTGGATTTGA |
| *LAMC1-F* | ATTTCAATCAACCGCTCT |
| *LAMC1-R* | GTTATGGACCTCCTTCGT |
| *CADM1-F* | TTTGAAGGACAGCAGGTTTCA |
| *CADM1-R* | AGGACTGTGATGGTGGTGTAACT |
| *HER3-F* | GTCTGTGTGACCCACTGCAACT |
| *HER3-R* | GGGTGGCAGGAGAAGCATT |
| *MYCBP-F* | TGGCACCTGTTGGAGACTATG |
| *MYCBP-R* | CACCAGCCATAGCCACATTC |
| *SNAI1-F* | GGTTCTTCTGCGCTACTGCTG |
| *SNAI1-R* | GTCGTAGGGCTGCTGGAAGG |
| *YAP1-F* | TGACCCTCGTTTTGCCATGA |
| *YAP1-R* | GTTGCTGCTGGTTGGAGTTG |
| *CD147-F* | CCGCAACCACCTTACTCG |
| *CD147-R* | GGACAGAGGTTTGGATGGTG |
| *SMAD4-F* | CCCATCCTGGACATTACTGG |
| *SMAD4-R* | TACACCAGTCCGTCCCTTTC |
| *ESR1-F* | TGGGCTTACTGACCAACCTG |
| *ESR1-R* | CCTGATCATGGAGGGTCAAA |
| *ZEB2-F* | AAGGAGCAGGTAATCGCAAG |
| *ZEB2-R* | GGAACCAGAATGGGAGAAACG |
