# Supplementary material for: miR-22 and miR-205 Drive Tumor Aggressiveness of Mucoepidermoid Carcinomas of Salivary Glands
Source: Front Oncol. 2022 Feb 9;11:786150. doi: 10.3389/fonc.2021.786150 (PMC8864291; doi:10.3389/fonc.2021.786150)
Supplement: Supplementary Table S2 — Differentially expressed miRNA in salivary gland mucoepidermoid carcinoma (MEC) compared to normal salivary gland (NSG) tissues. [file Table_2.docx]

**Supplementary Table S2.** Differentially expressed miRNA in salivary gland mucoepidermoid carcinoma (MEC) compared to normal salivary gland (NSG) tissues.

| **miRNA** | **miRBase ID** | **P-value** | **FDR** | **FC** |
| --- | --- | --- | --- | --- |
| miR-21-5p | MIMAT0000076 | < 1e-07 | 5E-05 | 10.2 |
| miR-21-3p | MIMAT0004494 | 5E-07 | 1E-04 | 6.7 |
| miR-625-5p | MIMAT0003294 | 3E-06 | 5E-04 | -18.5 |
| miR-205-3p | MIMAT0009197 | 4E-06 | 5E-04 | 14.7 |
| miR-885-5p | MIMAT0004947 | 5E-06 | 5E-04 | -10.7 |
| miR-892b | MIMAT0004918 | 8E-06 | 7E-04 | -2.7 |
| miR-1288-3p | MIMAT0005942 | 1E-05 | 1E-03 | -2.7 |
| miR-363-3p | MIMAT0000707 | 2E-05 | 1E-03 | -16.3 |
| miR-1305 | MIMAT0005893 | 2E-05 | 1E-03 | -2.4 |
| miR-139-3p | MIMAT0004552 | 3E-05 | 2E-03 | -3.9 |
| miR-181a-5p | MIMAT0000256 | 6E-05 | 2E-03 | 2.9 |
| miR-501-5p | MIMAT0002872 | 6E-05 | 2E-03 | -2.6 |
| miR-338-3p | MIMAT0000763 | 6E-05 | 2E-03 | -5.7 |
| miR-205-5p | MIMAT0000266 | 7E-05 | 3E-03 | 6.3 |
| miR-148a-3p | MIMAT0000243 | 8E-05 | 3E-03 | -13.5 |
| miR-324-3p | MIMAT0000762 | 9E-05 | 3E-03 | -2.1 |
| miR-22-3p | MIMAT0000077 | 2E-04 | 5E-03 | 2.0 |
| miR-145-5p | MIMAT0000437 | 2E-04 | 5E-03 | -4.4 |
| miR-625-3p | MIMAT0004808 | 2E-04 | 7E-03 | -3.3 |
| miR-224-3p | MIMAT0009198 | 3E-04 | 7E-03 | 5.3 |
| miR-3125 | MIMAT0014988 | 3E-04 | 8E-03 | -2.7 |
| miR-135a-5p | MIMAT0000428 | 4E-04 | 9E-03 | -5.1 |
| miR-1261 | MIMAT0005913 | 4E-04 | 9E-03 | 6.3 |
| miR-145-3p | MIMAT0004601 | 5E-04 | 1E-02 | -4.2 |
| miR-564 | MIMAT0003228 | 6E-04 | 1E-02 | -3.3 |
| miR-375 | MIMAT0000728 | 6E-04 | 1E-02 | -30.4 |
| miR-582-5p | MIMAT0003247 | 7E-04 | 1E-02 | -5.5 |
| miR-181c-3p | MIMAT0004559 | 9E-04 | 2E-02 | 3.2 |
| miR-3616-3p | MIMAT0017996 | 9E-04 | 2E-02 | -4.0 |
| miR-450a-5p | MIMAT0001545 | 1E-03 | 2E-02 | 3.6 |
| miR-30c-2-3p | MIMAT0004550 | 1E-03 | 2E-02 | -2.5 |
| miR-574-3p | MIMAT0003239 | 2E-03 | 3E-02 | -2.3 |
| miR-670-5p | MIMAT0010357 | 2E-03 | 3E-02 | 5.0 |
| miR-132-3p | MIMAT0000426 | 2E-03 | 3E-02 | 2.6 |
| miR-4324 | MIMAT0016876 | 2E-03 | 3E-02 | -4.0 |
| miR-455-3p | MIMAT0004784 | 2E-03 | 3E-02 | 6.3 |
| miR-500a-5p | MIMAT0004773 | 2E-03 | 3E-02 | -3.0 |
| ebv-miR-BART12 | MIMAT0003423 | 2E-03 | 3E-02 | 4.2 |
| miR-214-5p | MIMAT0004564 | 3E-03 | 3E-02 | 5.3 |
| miR-542-5p | MIMAT0003340 | 3E-03 | 3E-02 | 2.4 |
| miR-338-5p | MIMAT0004701 | 3E-03 | 4E-02 | -2.1 |
| miR-365a-3p | MIMAT0000710 | 3E-03 | 4E-02 | -2.2 |
| miR-224-5p | MIMAT0000281 | 4E-03 | 5E-02 | 5.3 |
| miR-3663-3p | MIMAT0018085 | 4E-03 | 5E-02 | 3.0 |
| miR-192-5p | MIMAT0000222 | 4E-03 | 5E-02 | -2.1 |
| miR-186-5p | MIMAT0000456 | 4E-03 | 5E-02 | -2.2 |

FC: Fold Change (MEC/NSG), FDR: False Discovery Rate.

This file contains a table displaying signifantly up and downregulated miRNAs in MEC compared to normal salivary glands (control). MiRBase IDs, fold changes, false discovery rate and P values are also shown.
